# Hypoxia-associated spontaneous pulmonary metastasis in human melanoma xenografts: involvement of microvascular hot spots induced in hypoxic foci by interleukin 8

**DOI:** 10.1038/sj.bjc.6600052

**Published:** 2002-01-21

**Authors:** E K Rofstad, E F Halsør

**Affiliations:** Group of Radiation Biology and Tumor Physiology, Department of Biophysics, Institute for Cancer Research, The Norwegian Radium Hospital, N-0310 Oslo, Norway

**Keywords:** melanoma, hypoxia, metastasis, angiogenesis, vascular hot spots, IL-8, VEGF

## Abstract

The aim of this study was to investigate whether tumour hypoxia and/or vascular hot spots promote the development of metastatic disease. The D-12 human melanoma xenograft line was used as a tumour model. Hypoxia and vascular hot spots were detected by immunohistochemistry using pimonidazole as a hypoxia marker and anti-CD31 antibody to visualize endothelial cells. Vascular hot spots were found to be induced in hypoxic foci, owing to hypoxia-induced up-regulation of angiogenesis stimulatory factors. This effect was mediated by interleukin 8 and possibly also by vascular endothelial growth factor. Interleukin 8 positive foci showed a high degree of co-localization with hypoxic foci, as revealed by immunohistochemistry. The incidence of spontaneous pulmonary metastases was associated with the density of hypoxic foci, the density of interleukin 8 positive foci and the density of vascular hot spots in the primary tumour. Treatment with neutralizing antibody against interleukin 8 and/or vascular endothelial growth factor resulted in hypoxia-induced necrosis rather than hypoxia-induced vascular hot spots and inhibited metastasis. Our study suggests a cause-effect relationship between hypoxia and metastasis in cancer and hence an elevated probability of metastatic disease in patients having primary tumours characterized by high densities of hypoxic foci and vascular hot spots.

*British Journal of Cancer* (2002) **86**, 301–308. DOI: 10.1038/sj/bjc/6600052 www.bjcancer.com

© 2002 The Cancer Research Campaign

## 

Most human tumours develop a pathophysiological microenvironment during growth, characterized by an irregular microvascular network and regions of chronically and transiently hypoxic cells (Vaupel *et al*, 1989). Side by side with the generation of an abnormal microenvironment, tumours gradually acquire aggressive phenotypic traits with time, a process termed malignant progression (Nowell, 1986). The final stage of the malignant progression is the development of cell variants showing invasive growth in surrounding normal tissues and metastatic spread to regional and distant organ sites. There is significant evidence that the microenvironment may accelerate the malignant progression of tumours and promote the development of metastatic disease (Rofstad, 2000). Thus, some tumour cells exposed to hypoxia *in vitro* show increased lung colonization efficiency after intravenous inoculation in mice (Young *et al*, 1988; Rofstad and Danielsen, 1999). Moreover, clinical studies have demonstrated associations between incidence of metastases and extent of hypoxia in the primary tumour in soft tissue sarcoma (Brizel *et al*, 1996) and cervical carcinoma (Höckel *et al*, 1996; Rofstad *et al*, 2000). These clinical studies, however, do not necessarily implicate a cause–effect relationship between hypoxia and metastasis. An alternative interpretation is that poor oxygenation is a secondary effect of tumour aggressiveness, i.e. the most aggressive cell phenotypes develop the most hypoxic primary tumours. There is also significant evidence that tumour hypoxia may be of limited significance in cancer metastasis (Rofstad, 2000). Thus, clinical studies involving several histological types of cancer have demonstrated associations between incidence of metastases and microvessel density in vascular hot spots of the primary tumour (Weidner, 1995). These studies are apparently inconsistent with those showing associations between hypoxia and metastasis, because tumour hypoxia is expected to be partly a result of poor oxygen supply owing to inadequate neovascularization (Vaupel *et al*, 1989). It has been hypothesized, however, that vascular hot spots may result from hypoxia-induced neovascularization mediated by angiogenesis stimulatory factors that are being up-regulated during hypoxia (Rofstad, 2000). The human melanoma xenograft study reported in the present communication supports this hypothesis and demonstrates further an association between spontaneous pulmonary metastasis on the one hand and hypoxia and vascular hot spots in the primary tumour on the other. The hypoxia-induced neovascularization was found to be mediated by interleukin 8 (IL-8), a multifunctional cytokine that shows potent angiogenic activities *in vitro* and *in vivo* (Bar-Eli, 1999), and possibly by vascular endothelial growth factor (VEGF), a strong specific mitogen for endothelial cells that also stimulates endothelial cell migration and reorganization (Ferrara and Davis-Smyth, 1997). The present communication also indicates a cause–effect relationship between hypoxia and metastasis in cancer, because the primary tumours in our study were initiated from the same monolayer cell culture of D-12 melanoma cells.

## MATERIALS AND METHODS

### Cell line

The experiments were performed with the human melanoma cell line D-12 (Rofstad, 1994). The cells were maintained in monolayer culture in RPMI-1640 (25 mM HEPES and L-glutamine) supplemented with 13% bovine calf serum, 250 mg l^−1^ penicillin and 50 mg l^−1^ streptomycin. The cultures were incubated at 37°C in a humidified atmosphere of 5% CO_2_ in air and subcultured twice a week. The cell line was verified to be free from *Mycoplasma* contamination.

### Hypoxia treatment

Monolayer cell cultures were exposed to hypoxia by using the steel-chamber method. The steel chambers were flushed with a humidified, highly purified gas mixture consisting of 95% N_2_ and 5% CO_2_ at a flow rate of 5 l min^−1^. The concentration of O_2_ in the medium was <10 p.p.m. after 30 min of flushing. Details of the procedure have been published elsewhere (Rofstad and Danielsen, 1999).

### VEGF and IL-8 expression *in vitro*

The expression of VEGF and IL-8 in D-12 cells was studied by Western blotting using standard experimental procedures (Rofstad and Halsør, 2000). Membranes were incubated with anti-human VEGF rabbit polyclonal antibody (Santa Cruz Biotechnology, Santa Cruz, CA, USA) or anti-human IL-8 mouse monoclonal antibody (R&D Systems, Abingdon, UK). Bound antibody was detected by a biotin–streptavidine alkaline phosphatase staining procedure. Recombinant human VEGF or IL-8 was used as positive control. The specificity of the antibody–antigen interactions was confirmed by peptide competition studies and by incubation of membranes in solutions without primary antibody.

### VEGF and IL-8 secretion *in vitro*

The secretion of VEGF and IL-8 in D-12 cells was studied by ELISA as described in detail elsewhere (Rofstad and Danielsen, 1999). Medium samples were collected from aerobic cultures in exponential growth and cultures exposed to hypoxia. Commercial ELISA kits (Quantikine; R&D Systems, Abingdon, UK) were used according to the instructions of the manufacturer to measure the concentrations of VEGF and IL-8 in the medium samples. Rate of protein secretion (*R*_sec_) was calculated as:


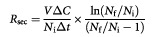


where Δ*C* is the increase in protein concentration during the time interval Δt. *N*_i_ and *N*_f_ are the initial and final cell numbers and *V* is the volume of medium. The second factor of the product is based on the assumption that the cell number increased exponentially with time during Δ*t*, an assumption that was verified to be valid for aerobic cultures. There was no cell proliferation under hypoxic conditions, i.e. the second factor of the product was approximately 1 for hypoxic cultures.

### Mice and tumours

Adult (8–10 weeks of age) female BALB/c-*nu/nu* mice were used as host animals for xenografted tumours. The mice were bred at our institute and maintained under specific pathogen-free conditions at constant temperature (24–26°C) and humidity (30–50%). Sterilized food and tap water were given *ad libitum*. Tumours were initiated from exponentially growing aerobic monolayer cell cultures. Approximately 3.5×10^5^ cells suspended in 10 μl of Ca^2+^ and Mg^2+^-free Hanks' balanced salt solution (HBSS) were inoculated intradermally into the flanks of mice. The growth and histological appearance of the tumours have been described elsewhere (Rofstad, 1994). Animal experiments were approved by the Institutional Committee on Research Animal Care and were performed according to the ethical standards of the UKCCCR ‘Guidelines for the Welfare of Animals in Experimental Neoplasia’.

### VEGF and IL-8 expression *in vivo*

The expression of VEGF and IL-8 in D-12 tumours was studied by immunohistochemistry using an indirect immunoperoxidase method (Rofstad and Halsør, 2000). Tumours were cut into 1-mm-thick slices that were fixed in phosphate-buffered 4% paraformaldehyde or snap-frozen in liquid nitrogen. Anti-human VEGF rabbit polyclonal antibody (Santa Cruz Biotechnology, Santa Cruz, CA, USA) or anti-human IL-8 rabbit polyclonal antibody (Endogen, Woburn, MA, USA) was used as primary antibody. Controls included omission of the primary antibody, incubation with normal rabbit immunoglobulin or normal rabbit serum and incubation with blocking peptides before staining. The sections were counterstained with haematoxylin. Quantitative analyses were based on eight cross-sections of each tumour.

### Tumour hypoxia

Pimonidazole {1-[(2-hydroxy-3-piperidinyl)propyl]-2-nitroimidazole} was used as a marker of tumour hypoxia. Hypoxic tumour regions were detected immunohistochemically as reported earlier by using a peroxidase-based indirect staining method (Rofstad and Måseide, 1999). Pimonidazole hydrochloride, kindly supplied by Professor JA Raleigh, was dissolved into 0.9% NaCl and administered i.p. to tumour-bearing mice in doses of 30 mg kg^−1^ body weight. The tumours were dissected free from the mice 4 h after the pimonidazole administration and 1-mm-thick tumour slices were fixed in phosphate-buffered 4% paraformaldehyde. Slides with tumour tissue preparations were incubated with polyclonal rabbit antiserum to pimonidazole–protein adducts, a kind gift from Professor JA Raleigh. Visualization of the antibody complex was achieved with the 3,3-diaminobenzidine chromogen. Haematoxylin was used for counterstaining. Eight cross-sections of each tumour were subjected to quantitative analyses. Area fractions showing pimonidazole staining and area fractions of necrosis were determined by image analysis as described elsewhere (Rofstad and Måseide, 1999).

### Tumour vascularization

The microvasculature of tumours was detected by immunohistochemistry using an avidin–biotin peroxidase-based staining method. Histological sections were prepared from 1-mm-thick tumour slices snap-frozen in liquid nitrogen. Anti-mouse CD31 rat monoclonal antibody (Research Diagnostics, Flanders, NJ, USA) was used as primary antibody. The 3,3-diaminobenzidine chromogen was used to visualize endothelial cells. Controls included omission of the primary antibody and incubation with blocking peptide before staining. The preparations were counterstained with haematoxylin. Quantitative analyses were based on eight cross-sections of each tumour. Microvessel density was scored by defining single, countable microvessels as defined by Weidner (1995).

### Metastasis assay

Primary tumours were initiated in the left flank of mice from monolayer cell cultures as described above. The inoculations were performed 24 h after the mice had been immunosuppressed by 5.0 Gy of whole body irradiation (Rofstad and Halsør, 2000). D-12 primary tumours suppress the growth of pulmonary micrometastases by secreting large quantities of the angiogenesis inhibitor thrombospondin-1 (Rofstad and Graff, 2001). The primary tumours were removed surgically when the largest diameter had attained a predetermined size to allow pulmonary micrometastases to develop into macroscopic metastases. The mice were examined twice a week for clinical signs of metastases, i.e. listlessness, weight loss or hunched posture. They were killed by cervical dislocation 3 months after the primary tumour was removed or when moribund. The lungs were examined for the presence of macroscopic metastases as described elsewhere (Rofstad and Halsør, 2000). Metastases were always found in moribund mice.

### Treatment with neutralizing antibody

The specific roles of VEGF and IL-8 in tumour metastasis were investigated by treating host mice with neutralizing antibody. The antibodies used for treatment were anti-human VEGF mouse monoclonal antibody (R&D Systems, Abingdon, UK) and anti-human IL-8 mouse monoclonal antibody (R&D Systems, Abingdon, UK). Antibody solutions were diluted in PBS and administered in volumes of 0.25 ml by i.p. injection. The treatments were given during the last 8 days before primary tumour removal and consisted of daily doses of 25 μg of anti-VEGF antibody, 100 μg of anti-IL-8 antibody, or both 25 μg of anti-VEGF antibody and 100 μg of anti-IL-8 antibody. Antibody treatment had no effect on tumour blood flow, as determined in separate experiments by using the ^86^Rb uptake method.

### Statistical analysis

Data are presented as arithmetic mean±s.e.m. Correlations between two parameters were searched for by linear regression analysis. Statistical comparisons of data were performed by using the Student's *t*-test (single comparisons) or by one-way ANOVA (multiple comparisons) under conditions of normality and equal variance. Under other conditions, comparisons were performed by non-parametric analysis using the Mann–Whitney rank sum test (single comparisons) or the Kruskal–Wallis one-way ANOVA on ranks (multiple comparisons). The Bonferroni's method (parametric tests) or the Dunnett's method (non-parametric tests) was used to identify data sets that differed from control data. Probability values of *P*<0.05 were considered significant. All *P*-values were determined from two-sided tests. The statistical analysis was performed by using the SigmaStat statistical software (Jandel Scientific GmbH, Erkrath, Germany).

## RESULTS

### Expression of VEGF and IL-8 *in vitro*

D-12 cells showed moderate secretion of VEGF and high secretion of IL-8 *in vitro*, as revealed by ELISA analysis. The rate of VEGF secretion was higher under hypoxic conditions (*R*_sec_=121±21 pg/(h·10^6^ cells); *n*=5) than under aerobic conditions (*R*_sec_=18±1 pg/(h·10^6^ cells); *n*=5) by a factor of approximately 7 (*P*=0.0012). Similarly, the rate of IL-8 secretion was higher under hypoxic conditions (*R*_sec_=1128±184 pg/(h·10^6^ cells); *n*=5) than under aerobic conditions (*R*_sec_=291±86 pg/(h·10^6^ cells); *n*=5) by a factor of approximately 4 (*P*=0.0033). Western blot analysis revealed that D-12 cells showed significant expression of VEGF and IL-8 *in vitro* and that the expression of both angiogenesis factors was up-regulated under hypoxic conditions (data not shown), consistent with the ELISA data.

### Hypoxia, vascularization and expression of VEGF and IL-8 *in vivo*

**Figure 1:**
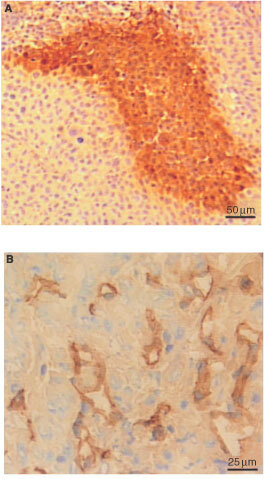
Immunohistochemical preparations of a D-12 tumour. An avidin-biotin peroxidase-based method was used for staining. Haematoxylin was used for counterstaining. Hypoxic focus visualized by anti-pimonidazole antibody staining (**A**) and section of a vascular hot spot visualized by anti-CD31 antibody staining (**B**).

Hypoxic pattern, vascular organization and the expression of VEGF and IL-8 in D-12 tumours were studied by immunohistochemistry, using tumours with diameters of approximately 10 mm. The tumours showed highly heterogeneous staining for pimonidazole, consistent with staining of chronically hypoxic cells without staining of normoxic or acutely hypoxic cells, as described earlier (Rofstad and Måseide, 1999). High-power examination of stained regions showed the staining to be discrete and mainly intracellular. Staining was seen primarily in the cytoplasm, but prevalent intranuclear staining also occurred frequently. The boundary line between stained and unstained cells was remarkably sharp, i.e. the cells showed either intense brown staining or no staining. Thus, hypoxic cells could easily be distinguished from normoxic cells. Necrotic regions were always encompassed by a rim of hypoxic cells, 2–4 cell layers thick, as illustrated elsewhere (Rofstad and Måseide, 1999). Moreover, foci of hypoxic cells scattered throughout the tissue were seen in tumour regions without necrosis (Figure 1A

). The shape and size of the hypoxic foci differed substantially. Hypoxic foci without necrosis had a longest diameter of up to 800 μm.

The tumours showed highly heterogeneous staining also for CD31, consistent with staining of endothelial cells. Isolated microvessels as well as clusters of microvessels, i.e. vascular hot spots, were seen in tumour regions without necrosis. High-power inspection of vascular hot spots revealed functional microvessels, i.e. lumens with erythrocytes encircled by endothelial cells, and individual endothelial cells separated by parenchymal melanoma cells (Figure 1B). CD31-positive mitotic figures were seen in some vascular hot spots. The vascular hot spots differed substantially in shape and size, showed a longest diameter of up to 800 μm, and were seen in non-necrotic tissue in both central and peripheral tumour regions. The microvessel density was 5–10-fold higher within vascular hot spots than elsewhere in non-necrotic tissue.

**Figure 2:**
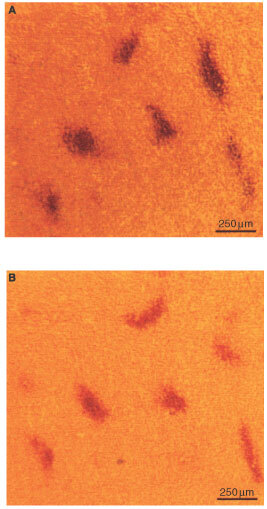
Immunohistochemical preparations of adjacent sections of a D-12 tumour. An avidin-biotin peroxidase-based method was used for staining. Haematoxylin was used for counterstaining. IL-8 positive foci visualized by anti-IL-8 antibody staining (**A**) and hypoxic foci visualized by anti-pimonidazole antibody staining (**B**).

Moreover, the tumours showed significant staining for both VEGF and IL-8. The VEGF expression was relatively homogeneous within tumours, but was slightly up-regulated nearby some necrotic regions, as illustrated previously (Danielsen and Rofstad, 2000). The VEGF staining appeared to be localized in the cytoplasm of the cells. Very few negative cells were observed. The IL-8 expression, on the other hand, was highly heterogeneous. The IL-8 staining was primarily intracellular and the staining pattern was similar to that for pimonidazole. Examination of adjacent sections revealed a high degree of co-localization of IL-8 and pimonidazole staining (Figure 2

). Thus, necrotic regions were always surrounded by a rim of IL-8 positive cells. These rims were usually slightly broader than the corresponding hypoxic rims. Moreover, foci of IL-8 positive cells were seen in tumour regions without necrosis. When IL-8 positive foci were seen, hypoxic foci were always seen in the same positions in the adjacent section. The degree of co-localization the reversed way differed among individual tumours. When hypoxic foci were seen, IL-8 positive foci were seen in the same positions in the adjacent section in 84–97% of the cases. The IL-8 positive foci were generally slightly larger than the hypoxic foci (Figure 2), and the boundary line between stained and unstained cells was not so sharp as for pimonidazole staining. There was no significant co-localization of IL-8 positive foci and vascular hot spots.

### Spontaneous metastasis

**Figure 3:**
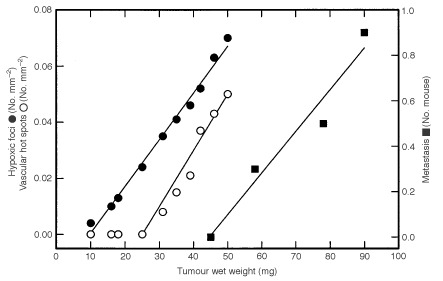
Density of hypoxic foci in the primary tumour (•), density of vascular hot spots in the primary tumour (○) and number of pulmonary metastases in the host (▪) *vs* wet weight of the primary tumour. The densities of hypoxic foci and vascular hot spots were determined in one experiment and the number of pulmonary metastases in another. Points represent single D-12 tumours (•, ○) or mean values of 10 D-12 tumours/mice (▪).

The frequency of metastatic dissemination as well as the densities of hypoxic foci, IL-8 positive foci and vascular hot spots in the primary tumour were determined as a function of time after tumour initiation to investigate whether vascular hot spots developed from hypoxic foci and whether the development of hypoxic foci/vascular hot spots preceded metastatic dissemination. Ten mice with small D-12 tumours were included in the first experiment. The primary tumours were removed when the wet weights were within the range of 10–50 mg and subjected to histological examinations. Only vascular hot spots with a longest diameter within the range of 400–800 μm were included in the measurements to ensure correct hot spot identification. The measurements of the density of hypoxic foci were for the sake of comparison based on hypoxic foci with a longest diameter within the same range. As IL-8 positive foci in general were larger than the corresponding hypoxic foci, the density of IL-8 positive foci was determined by scoring foci with a longest diameter of up to 1200 μm. The densities of hypoxic foci and vascular hot spots are plotted *vs* tumour wet weight in Figure 3

. Hypoxic foci were only just seen in the 10-mg tumour and then the density increased linearly with the wet weight. Vascular hot spots could not be detected in tumours with wet weights below 25 mg. For larger tumours, the density of vascular hot spots increased linearly with the wet weight with a slope similar to that for the density of hypoxic foci. The delay in the development of vascular hot spots relative to the development of hypoxic foci corresponded to a growth period of 3–4 days. There was an even higher degree of co-localization of IL-8 and pimonidazole staining in these small tumours than in the larger tumours described above; the density of IL-8 positive foci increased with the tumour wet weight in the same way as the density of hypoxic foci (data not shown). These small tumours showed relatively homogeneous expression of VEGF, similar to the larger tumours described above. None of the 10 host mice developed pulmonary metastases during the 3-month follow-up period, suggesting that the development of hypoxic foci/vascular hot spots precedes metastatic dissemination in D-12 tumours. This was investigated further in a second experiment involving four groups of 10 mice each. The primary tumours were removed when the wet weights had attained approximately 45 mg (group 1), approximately 60 mg (group 2), approximately 75 mg (group 3) or approximately 90 mg (group 4). Pulmonary metastases developed in 0 of 10 mice (group 1), 2 of 10 mice (groups 2 and 3) and 3 of 10 mice (group 4). A plot of the mean number of metastases per mouse *vs* mean tumour wet weight for the four groups is included in Figure 3. These metastasis data together with the hypoxic foci/vascular hot spots data from the first experiment give strong evidence that metastatic dissemination is preceded by the development of hypoxic foci/vascular hot spots in D-12 tumours. Moreover, the primary tumours of group 1 were subjected to histological examinations. The densities of hypoxic foci and vascular hot spots (No. mm^−2^) were determined to be 0.062±0.003 and 0.039±0.002, respectively, consistent with the hypoxic foci/vascular hot spots data in Figure 3.

**Figure 4:**
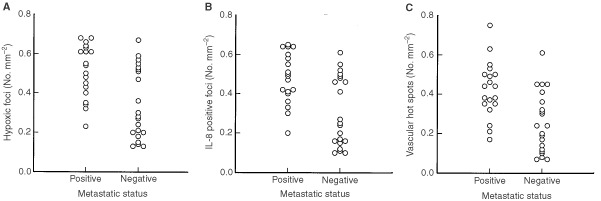
Hypoxia, IL-8 expression and neovascularization in metastatic and non-metastatic D-12 primary tumours. Points represent single tumours. Density of hypoxic foci (**A**), density of IL-8 positive foci (**B**) and density of vascular hot spots (**C**).

A third experiment involving 40 mice was performed to investigate whether tumour hypoxia, IL-8 expression and/or tumour neovascularization were associated with spontaneous pulmonary metastasis in D-12 tumours. The primary tumours were removed when the largest diameter had attained approximately 10 mm and the following parameters were determined and related to the metastatic status of the hosts: hypoxic fraction, density of hypoxic foci, normoxic fraction, necrotic fraction, IL-8 positive fraction, density of IL-8 positive foci, hot spot microvessel density and density of vascular hot spots. Hypoxic foci, IL-8 positive foci and vascular hot spots were defined as described above. Nineteen mice developed metastases whereas 21 mice did not. The primary tumours that gave rise to metastases showed an approximate 1.5-fold higher density of hypoxic foci than those that did not give rise to metastases (Figure 4A

; *P*=0.0021). Similarly, the hypoxic fraction (*HF*), calculated from the total area of tissue that stained positive for pimonidazole (including hypoxic tissue adjacent to necrosis and hypoxic foci of all sizes) and the total tissue area, was higher in the primary tumours that developed metastases (*HF*=14±1%) than in those that did not develop metastases (*HF*=11±1%) by a factor of approximately 1.3 (*P*=0.041). Neither the normoxic fraction (*NOF*) nor the necrotic fraction (*NF*) differed significantly between metastatic (*NOF*=63±4%; *NF*=23±3%) and non-metastatic (*NOF*=64±5%; *NF*=25±4%) primary tumours (*P*>0.05 for both *NOF* and *NF*). Moreover, the primary tumours that metastasized showed an approximate 1.5-fold higher density of IL-8 positive foci than those that did not metastasize (Figure 4B; *P*=0.0015). Also the IL-8 positive fraction (*IPF*), calculated from the total area of tissue that stained positive for IL-8 (including IL-8 positive tissue adjacent to necrosis and IL-8 positive foci of all sizes) and the total tissue area, was higher in the primary tumours that formed metastases (*IPF*=26±2%) than in those that did not form metastases (*IPF*=17±2%) by a factor of approximately 1.5 (*P*=0.0030). Finally, the metastatic primary tumours showed an approximate 1.6-fold higher density of vascular hot spots than those that did not metastasize (Figure 4C; *P*=0.0010). The hot spot microvessel density (*MVD* (No. mm^−2^)), on the other hand, did not differ significantly between metastatic (*MVD*=118±12) and non-metastatic (*MVD*=123±11) primary tumours (*P*>0.05). It should be noticed that although the density of hypoxic foci, IL-8 positive foci and vascular hot spots differed significantly between metastatic and non-metastatic primary tumours, a substantial fraction of the non-metastatic tumours showed density values for hypoxic foci, IL-8 positive foci and vascular hot spots within the same ranges as those for the metastatic tumours (Figure 4).

**Figure 5:**
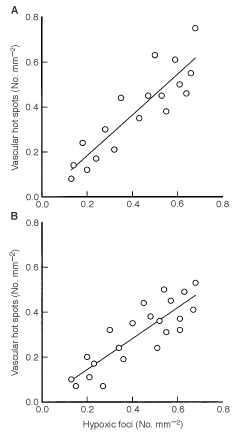
Density of vascular hot spots *vs* density of hypoxic foci in D-12 tumours. Points represent single tumours. Tumours with necrotic fractions below 25% (**A**) and tumours with necrotic fractions within the range of 25–50% (**B**).

The density of vascular hot spots was positively correlated to the density of hypoxic foci in these primary tumours. Different correlations were found for tumours with low necrotic fractions, i.e. necrotic fractions below 25% (Figure 5A

; *R*^2^=0.80, *P*<0.00001), and tumours with high necrotic fractions, i.e. necrotic fractions within the range of 25–50% (Figure 5B; *R*^2^=0.72, *P*<0.00001). The slope of the regression curve in Figure 5A was not significantly different from unity (*P*>0.05), but was significantly higher than that of the regression curve in Figure 5B (*P*=0.045).

### Treatment with neutralizing antibody

**Figure 6:**
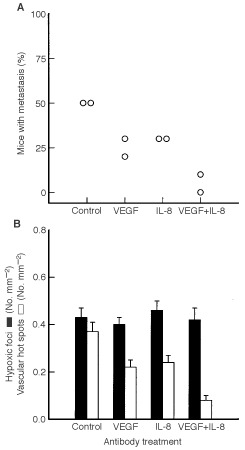
Effects of anti-VEGF treatment, anti-IL-8 treatment, and combined anti-VEGF and anti-IL-8 treatment on development of hypoxia, neovascularization and spontaneous pulmonary metastasis in D-12 tumours. (**A**) Percentage of mice with metastasis. Points represent single experiments involving 10 mice each. (**B**) Densities of hypoxic foci and vascular hot spots. Columns represent mean values of 20 mice. Bars represent s.e.m.

The involvement of VEGF and IL-8 in spontaneous pulmonary metastasis in D-12 melanoma was investigated by treating host mice with neutralizing antibody against VEGF, IL-8, or both VEGF and IL-8 (Figure 6A

). Ten of 20 untreated control mice developed metastases. The incidence of metastases was reduced after anti-VEGF treatment (*P*=0.022), anti-IL-8 treatment (*P*=0.048), and combined anti-VEGF and anti-IL-8 treatment (*P*=0.0025). Moreover, the incidence of metastases was lower after combined anti-VEGF and anti-IL-8 treatment than after anti-VEGF treatment alone (*P*=0.048) or anti-IL-8 treatment alone (*P*=0.022).

The density of hypoxic foci, the density of vascular hot spots and necrotic fraction were scored in these primary tumours to investigate whether hypoxic foci and vascular hot spots were involved in the development of pulmonary metastases in D-12 melanoma (Figure 6B). The control group showed densities of hypoxic foci and vascular hot spots that were not significantly different (*P*>0.05). The density of hypoxic foci was not influenced significantly by anti-VEGF treatment (*P*>0.05), anti-IL-8 treatment (*P*>0.05), or combined anti-VEGF and anti-IL-8 treatment (*P*>0.05). The density of vascular hot spots, on the other hand, was reduced after anti-VEGF treatment (*P*=0.0028), anti-IL-8 treatment (*P*=0.012), and combined anti-VEGF and anti-IL-8 treatment (*P*<0.00001). Moreover, the density of vascular hot spots was lower after combined anti-VEGF and anti-IL-8 treatment than after anti-VEGF treatment alone (*P*=0.0058) or anti-IL-8 treatment alone (*P*=0.0013). The control tumours showed a necrotic fraction (*NF*) of 22±3%. The necrotic fraction was increased after anti-VEGF treatment (*NF*=38±3%; *P*=0.0060), anti-IL-8 treatment (*NF*=35±4%; *P*=0.034), and combined anti-VEGF and anti-IL-8 treatment (*NF*=43±4%; *P*=0.00022).

## DISCUSSION

The incidence of spontaneous pulmonary metastases was associated with the extent of hypoxia in the primary tumour in D-12 melanoma. Clinical studies of soft tissue sarcoma (Brizel *et al*, 1996) and cervical carcinoma (Höckel *et al*, 1996; Rofstad *et al*, 2000) have also demonstrated associations between incidence of metastases and poor oxygenation of the primary tumour. From these clinical studies, it has not been possible to establish whether hypoxia causes metastasis or whether the most metastatic cell phenotypes develop the most hypoxic primary tumours. The D-12 primary tumours were initiated from the same culture of monolayer cells, suggesting a cause–effect relationship between hypoxia and metastasis in cancer.

D-12 primary tumours showed distinct hypoxic foci and distinct vascular hot spots in non-necrotic tissue. We propose the following hypothesis: a hypoxic focus develops into either a necrotic region or a vascular hot spot in D-12 tumours, depending on whether the hypoxia-induced up-regulation of angiogenesis stimulatory factors in the hypoxic focus is sufficiently high to stimulate adequate neovascularization. Our hypothesis is supported by several observations. First, small tumours developed hypoxic foci before they developed vascular hot spots. The delay in the development of vascular hot spots relative to the development of hypoxic foci was 3–4 days. Second, hypoxic foci and vascular hot spots showed similar size distributions and were scattered throughout non-necrotic tissue in similar patterns, independent of tumour size. Third, the density of vascular hot spots was positively correlated to the density of hypoxic foci in large tumours. The slope of the regression curve pertaining to tumours with low necrotic fractions was close to unity and significantly higher than that of the regression curve pertaining to tumours with high necrotic fractions. Moreover, hypoxia increased the expression and secretion of VEGF and IL-8 *in vitro*, and has been shown to stimulate D-12 induced angiogenesis *in vivo* (Rofstad and Danielsen, 1999). Treatment with neutralizing antibody against VEGF, IL-8, or VEGF and IL-8 *in vivo* did not change the density of hypoxic foci, but reduced the density of vascular hot spots and increased the necrotic fraction comparatively. The anti-VEGF and anti-IL-8 neutralizing antibodies used here have no antiproliferative or cytotoxic effects on D-12 cells in culture (Rofstad and Halsør, 2000). Most importantly, examination of immunohistochemical preparations of adjacent sections revealed a high degree of co-localization of IL-8 and pimonidazole staining in D-12 tumours; the expression of IL-8 was up-regulated in hypoxic foci and in hypoxic cells adjacent to necroses. Hypoxia-induced up-regulation of IL-8 has also been demonstrated by immunohistochemistry in pancreatic cancer xenografts (Shi *et al*, 1999) and human ovarian carcinomas (Xu *et al*, 1999). Up-regulation of VEGF in hypoxic foci, however, could not be detected in immunohistochemical preparations of D-12 tumours, perhaps because VEGF diffused readily from hypoxic tissue into surrounding normoxic tissue.

Hypoxia was found to promote spontaneous metastasis in D-12 melanoma; the hypoxic fraction and the density of hypoxic foci were higher in metastatic than in non-metastatic primary tumours. The density of hypoxic foci had a higher discriminative power than the hypoxic fraction, suggesting that the hypoxia-induced metastasis was primarily attributable to hypoxic foci in non-necrotic tissue rather than to hypoxic regions adjacent to necrosis. There is significant evidence that the mechanism by which metastasis was promoted by hypoxic foci involved hypoxia-induced up-regulation of angiogenesis stimulators and vascular hot spots induced in hypoxic foci. Thus, the densities of IL-8 positive foci and vascular hot spots were higher in metastatic than in non-metastatic primary tumours in untreated mice, and treatment with neutralizing antibody against VEGF, IL-8, or VEGF and IL-8 reduced the incidence of metastases and the density of vascular hot spots comparatively. Vascular hot spots are composed of immature capillaries and capillary sprouts that may facilitate tumour cell intravasation and hence metastasis by several mechanisms (Fidler and Ellis, 1994; Weidner, 1995). Immature capillaries are leaky and have fragmented basement membranes, making them more accessible to tumour cells than mature capillaries. Capillary sprouts are characterized by extensive endothelial cell proliferation and migration, and proliferating and migrating endothelial cells secrete paracrine growth factors and degradative enzymes that may advance the escape of tumour cells into the neovasculature. Finally, growing capillaries may actively promote intravasation by engulfing tumour cells.

The time course experiments suggest that the development of hypoxic foci and vascular hot spots was a necessary condition for metastatic dissemination in D-12 melanoma. The tumours started developing hypoxic foci and vascular hot spots when reaching wet weights of approximately 10 and 25 mg, respectively, whereas metastatic dissemination did not occur until a wet weight of 50–60 mg was reached, i.e. until a substantial number of hypoxic foci and vascular hot spots had developed. The presence of hypoxic foci and vascular hot spots, however, was not a sufficient condition for metastasis in D-12 melanoma. Although the densities of hypoxic foci and vascular hot spots differed significantly between metastatic and non-metastatic primary tumours, a substantial percentage of the non-metastatic tumours showed density values within the same ranges as those of the metastatic tumours. Consequently, metastatic disease can probably not be predicted from the densities of hypoxic foci and vascular hot spots in the primary tumour.

There is experimental evidence that the mechanism of the hypoxia-induced spontaneous metastasis in D-12 melanoma also involved increased probability of tumour cells trapped in the lung capillary bed to give rise to macroscopic growth, owing to hypoxia-induced VEGF up-regulation. It has been shown that D-12 cells exposed to hypoxia *in vitro* before being inoculated intravenously in mice have an increased lung colonization efficiency relative to aerobic control cells and that the increase can be prevented by treatment with anti-VEGF antibody (Rofstad and Danielsen, 1999). Hypoxia-induced VEGF up-regulation may promote the development of metastases from tumour cells trapped in a secondary organ by increasing the permeability of the capillary bed and hence facilitate tumour cell extravasation, enhancing the survival, migration and proliferation of extravasated tumour cells, and stimulating the initial phases of tumour angiogenesis in the secondary organ (Ferrara and Davis-Smyth, 1997; Rofstad and Danielsen, 1999; Li *et al*, 2000).

Some clinical studies have demonstrated associations between metastasis and hot spot microvessel density in the primary tumour (Weidner, 1995), whereas others have demonstrated associations between metastasis and tumour hypoxia (Brizel *et al*, 1996; Höckel *et al*, 1996; Rofstad *et al*, 2000). These two groups of studies have been considered to be incompatible, mainly because tumour hypoxia has been shown to be associated with poor tumour vascularization (Vaupel *et al*, 1989). Our study suggests a mechanism of hypoxia-induced metastasis that involves induction of vascular hot spots and thus unifies these clinical observations; tumour hypoxia leads to elevated secretion of hypoxia-inducible angiogenesis stimulators, elevated secretion of angiogenesis stimulators causes development of vascular hot spots, vascular hot spots promote tumour cell intravasation, and increased intravasation results in increased probability of metastasis. This mechanism does not necessarily involve VEGF or IL-8; other angiogenesis stimulators have also been shown to be up-regulated by hypoxia (Griffiths *et al*, 1997; Hartmann *et al*, 1999).

Two types of tumour hypoxia have been recognized: chronic or diffusion-limited hypoxia, arising from limitations in oxygen diffusion owing to oxygen consumption by the tumour cells, and acute or perfusion-limited hypoxia, resulting from transient cessations in microregional tumour blood flow (Horsman, 1995). Our study introduces a need for dividing the diffusion-limited hypoxia into two subcategories: permanent diffusion-limited hypoxia, i.e. diffusion-limited hypoxia resulting in cell death and development of necrotic regions, and transient diffusion-limited hypoxia, i.e. diffusion-limited hypoxia leading to angiogenesis and cell reoxygenation. Tumour oxygenation status was measured polarographically with needle electrodes in the clinical studies showing associations between metastasis and tumour hypoxia, and it was assumed that hypoxia-induced metastases arose from cells in tumour regions having been subjected to perfusion-limited hypoxia, mainly because such cells can be found adjacent to microvessels and thus can intravasate easily (Brizel *et al*, 1996; Höckel *et al*, 1996; Rofstad *et al*, 2000). However, perfusion-limited hypoxia is generally of short duration and may not necessarily induce significant gene up-regulation (Horsman, 1995). Our study on the other hand, suggests that hypoxia-induced metastases arise from cells in tumour region having been subjected to transient diffusion-limited hypoxia. Such cells can readily escape into the microvasculature as described above, and transient diffusion-limited hypoxia most likely lasts sufficiently long for maximum hypoxia-induced gene up-regulation to occur (Rofstad, 2000).

It should also be noted that the results of our study are not identical to those of the clinical studies having demonstrated associations between metastasis and hot spot microvessel density in the primary tumour (Weidner, 1995). We found an association between metastasis and density of vascular hot spots, but no association between metastasis and hot spot microvessel density as was found in the clinical studies. In fact, the mean hot spot microvessel density did not differ significantly among individual D-12 tumours, perhaps because they were derived from the same cell line and thus, in contrast to the clinical tumours, had the same genetic background. The density of vascular hot spots has not been determined in clinical specimens, but it is likely to be governed by mechanisms similar to those governing the hot spot microvascular density and may thus be correlated to this parameter.

The mechanism of hypoxia-induced metastasis reported here for D-12 melanoma is by no means the only mechanism by which tumour hypoxia may induce metastasis. A recent review, based on novel insights from studies of experimental tumours and cells in culture and recent advances in gene regulation and signal transduction, has identified several possible mechanisms of hypoxia-induced cancer metastasis (Rofstad, 2000). Thus, hypoxia may induce point mutations and DNA strand breakage leading to deletions, amplifications and genomic instability. Hypoxia may also provide a physiological pressure in tumours selecting for metastatic cell phenotypes. Moreover, hypoxia may induce a temporary increase in the expression of gene products involved in the metastatic cascade, either through gene amplifications or through normal physiological processes by activating oxygen sensors, hypoxia signal transduction pathways and DNA transcription factors (Rofstad, 2000).

In summary, tumour hypoxia induced spontaneous pulmonary metastasis in D-12 melanoma by a mechanism which involved induction of vascular hot spots by hypoxic foci, owing to hypoxia-induced up-regulation of IL-8 and possibly also of VEGF. Treatment with anti-VEGF and/or anti-IL-8 neutralizing antibody led to the development of necrosis rather than vascular hot spots and inhibited hypoxia-induced metastasis. Our study suggests a cause–effect relationship between hypoxia and metastasis in cancer and a poor prognosis of patients having primary tumours characterized by low oxygen tension.
